# The effects of an area-based intervention on the uptake of maternal and child health assessments in Australia: A community trial

**DOI:** 10.1186/1472-6963-9-53

**Published:** 2009-03-25

**Authors:** Margaret Kelaher, David Dunt, Peter Feldman, Andrea Nolan, Bridie Raban

**Affiliations:** 1 Centre for Health Policy, Programs and Economics, School of Population Health, University of Melbourne, Melbourne, Australia; 2 Faculty of Education, Deakin University, Melbourne, Australia; 3 Early Childhood Consortium, Faculty of Education, University of Melbourne, Melbourne, Australia

## Abstract

**Background:**

Recognition of the importance of the early years in determining health and educational attainment and promotion of the World Health Organization Health for All (HFA) principles has led to an international trend towards community-based initiatives to improve developmental outcomes among socio-economically disadvantaged children. In this study we examine whether, Best Start, an Australian area-based initiative to improve child health was effective in improving access to Maternal and Child Health (MCH) services.

**Methods:**

The study compares access to information, parental confidence and annual 3.5 year Ages and Stages visiting rates before (2001/02) and after (2004/05) the introduction of Best Start. Access to information and parental confidence were measured in surveys of parents with 3 year old children. There were 1666 surveys in the first wave and 1838 surveys in the second wave. The analysis of visiting rates for the 3.5 year Ages and Stages visit included all eligible Victorian children. Best Start sites included 1,739 eligible children in 2001/02 and 1437 eligible children in 2004/05. The comparable figures in the rest of the state were and 45, 497 and 45, 953 respectively.

**Results:**

There was a significant increase in attendance at the 3.5 year Ages and Stages visit in 2004/05 compared to 2001/02 in all areas. However the increase in attendance was significantly greater at Best Start sites than the rest of the state. Access to information and parental confidence improved over the course of the intervention in Best Start sites with MCH projects compared to other Best Start sites.

**Conclusion:**

These results suggest that community-based initiatives in disadvantaged areas may improve parents' access to child health information, improve their confidence and increase MCH service use. These outcomes suggest such programmes could potentially contribute to strategies to reduce child health inequalities.

## Background

Recognition of the importance of the early years in determining health and educational attainment has led to a number of specially designed, community-based initiatives to improve developmental outcomes among socio-economically disadvantaged children. [1,2] This has been accompanied by increasing evidence that living in disadvantaged areas is associated with worse health. [3-15] This has resulted in a number of initiatives which aim to improve health outcomes through the development of community and service provider partnerships as a way of increasing co-ordination between services. These initiatives aim to identify and address important gaps in service provision so as to better meet community needs. In so doing, they reflect a wider shift towards area-based interventions as part of the Health for All (HFA) principles promoted by the World Health Organization. [3]

Initiatives such as Sure Start in the UK and Best Start [4], among others, in Australia focus on innovations and extensions of services across a wide front. For example Sure Start offers outreach and home visiting services; support to families (parental support and advice); community health services (child health, women's health and general health) and good quality play, learning and childcare services. The evaluation of the program suggested that Sure Start areas were more effective than control areas in improving social development and increasing the use of child and family services [1].

These findings have occurred against a backdrop of mixed evidence for the health benefits of area-based initiatives more generally. [5] This may be due to the interventions themselves, measurement issues and mobility from areas. [5] Reviews of the evidence of the health impacts of area-based interventions have highlighted the need to focus on the pathways through which interventions would be expected to influence health. [6-8] Most research on these interventions has tended to focus on health or developmental outcomes rather than intermediate stages in the pathway through which interventions would be expected to affect health status. [1,5,8] These intervening steps, particularly around the use of early childhood services, are very important in terms of addressing health disadvantage in children where the deleterious effects of adverse exposures and health benefits of intervention may not show up till later in life.

In this paper we report on the evaluation of the Australian initiative, Best Start. The evaluation focussed the impact of interventions on access to health assessments services and parent's access to information and confidence. We aim to answer two questions:

1) Do the projects initiated by Best Start partnerships improve access to Maternal and Child Health (MCH) Services?

2) What are the mechanisms through which such changes might occur?

### Best Start

Best Start is an initiative of the Victorian Department of Human Services (DHS) in partnership with the Department of Education and Training (DE&T) and other departments of the Victorian state Government. Best Start aims to improve the health, development, learning and well-being of all young children across Victoria from pregnancy into early school years. There is also a particular focus on improving access to services in vulnerable and underserved groups.

Five Best Start demonstration sites were identified for funding in 2002 and an additional six sites were identified in 2003. Sites were identified across the state in metropolitan, regional and rural areas. Each site had a facilitator, funded through the program, and a partnership with representatives from state and local government, non-government agencies as well as local community groups and local parents. Projects, developed and delivered on behalf of the partnerships were largely designed to add value by increasing co-ordination, co-operation and linkages between existing services rather than introduce new services or expand existing services. [9]

DHS identified seven health outcome areas as well as four educational and two housing/child protection outcome areas that Best Start programs could choose to target. The Health and well-being indicator areas were Breastfeeding, Women smoking during pregnancy, Immunisation, Attendance at MCH, Attendance at hospital ED for specific conditions, Children's diet and physical activity and Community safety. The Education and schooling indicator areas were Parents reading to their children, Participation in preschool/kindergarten, Absences from primary school, Reading abilities. As a result, the portfolio of projects offered by Best Start sites varied considerably. The program including both government policy direction and implementation by partnerships is fully described in [4] and [9] respectively. The health outcome areas targeted by the projects were breast-feeding and attendance at MCH assessments. [9] In this paper we focus on attendance at MCH, specifically the 3.5 year Ages and Stages visit. [10].

### Maternal and Child Health Services

Developmental surveillance in Victoria is undertaken at key age and stages using a variety of tools which addresses communication, gross motor and fine motor skills, problem solving and personal and social issues. [11] Parents are also provided with a range of information about parenting, health issues and services. [10]

Victorian mothers are provided with one home visit shortly after the birth of their child. The visit is instigated by the local MCH nurse who is automatically contacted when a child is born. A meta-analysis of programs involving at least one post partum home visit suggest that these programs have a positive impact on developmental outcomes and home environment. [12,13] MCH assessments other than the initial home consultation are generally conducted at a MCH centre. All MCH visits are free. The 3.5 year Ages and Stages visit is seen as particularly critical because it enables intervention in developmental problems before school which can in turn reduce the severity and/or adverse effects associated with any delay in future development [14]. It is also seen as a key intervention point to encourage preschool participation which can also improve developmental outcomes. [15] The 3.5 year Ages and Stages visit was seen as an important target for Best Start because rates of participation are about half that for the initial home consultation. [9]

Strategies underlying interventions at Best Start sites included social marketing, cross-service promotion and co-ordination, reminders and the development of playgroups with a particular focus on targeting vulnerable and underserved groups. [9] To illustrate this, a few Best Start sites made structural changes to their MCH programs such as establishing new service arrangements (playgroups, community hubs and family resource centres). Most engaged in new programs of promotion and outreach to child care services and parent reading groups. These promotional activities particularly targeted Indigenous and immigrant groups. Bags with children's books, parenting information and welcome packs were used to promote services. There is good evidence from systematic reviews that reminder systems improve childhood immunisation rates in the order of 1 to 20 percentage points [16]. All types of reminders are effective [16]. It is therefore likely that social marketing activities which remind parents about the need to attend MCH services would have a similar effect on attendance at MCH assessments.

Parental confidence might also be a crucial variable in improving the uptake of health services. Recent evidence suggests that further evidence that parental confidence might be a major barrier to accessing health services[17]. There is growing evidence suggesting that parenting practices are associated with emotional and behavioural problems in children under 3 years[18,19] and may mediate the impact of socioeconomic position on child health.

## Methods

### Design

The study used a quasi-experimental design to assess changes in attendance rates at MCH before and after the introduction of Best Start projects at sites with MCH projects compared to the rest of the state. There was biannual reporting of projects occurring at each site over the course of the intervention. This enabled clear identification of the sites with and without MCH projects.

The sites were selected by DHS before the start of the study because of worse social characteristics and health outcomes than the rest of the state. [4] Intention to treat analysis was used for sites with projects, given that all eligible parents/children were targeted by the project.

The study also included more detailed survey of parents concerning the antecedents of their changes in service use including parental knowledge of MCH services and self-efficacy as parents. Surveys were conducted both before and after the introduction of Best Start projects using two cross-sectional samples of parents of three year old children. [20] The intervention group consisted of Best Start sites with MCH projects and the control group consisted of Best Start sites that had not implemented MCH projects.

The evaluation was approved by ethics committees at the Victorian Department of Human Services and the University of Melbourne.

### Instruments and procedures

#### MCH participation

Data for MCH participation is routinely collected from clinics, aggregated at LGA level and provided to DHS. Local clinic data was used when Best Start sites did not include the entire LGA. Denominators for MCH projects were based on the total number of children in each area, in each age group. Data was coded in Australian financial years (July 1 to June 30) and included the period from 2000–2001 to 2004/2005.

#### Parent's Survey

The parent's survey measured access to information and confidence in being a parent but do not include whether the parent was exposed to the Best Start MCH program or not or if they presented to the 3.5 year MCH visit or not. The questions were adapted from a number of well-established early childhood development instruments. [21-23]

The questionnaire was translated into the three most common community languages across Best Start sites (Turkish, Vietnamese and Cantonese). Translated surveys were then back translated for verification of the precision of the questions in relation to the original survey.

The questionnaire was sent to parents attached to the official form used to enrol a child for kindergarten in the following year. Distribution methods varied slightly between sites. A detailed description of the survey and its implementation is included in the evaluation report. [9]

### Sample

#### MCH participation

In 2001/02 there were 1,739 children eligible for their 3.5 year Ages and Stages visit in Best Start sites and 45, 497 in the rest of Victoria. In 2004/05 the numbers were 1437 and 45, 953 respectively.

#### Parent's survey

There were 1666 usable questionnaires returned in the first wave of data collection and 1838 in the second wave. While efforts were made to establish exact tallies of surveys sent/handed to parents by sites, this was difficult to achieve because of variation between sites. Response rates therefore are likely to underestimate actual return rates. The estimated response rate in the first wave was 37.3% assuming 25% wastage of forms. In the second wave where tally numbers were more accurately estimated, the response rate was estimated to be 34.9% (though this is still likely to be an underestimate).

Given this lack of precision about the relatively low response rate, it is important to demonstrate how representative the sample population was in terms of the whole population of parents and families of which it is a part. Table 1 compares the characteristics of parents and their families to the characteristics of the population based on LGA level data. The survey sample and the characteristics of the LGA were similar in terms of parents born overseas (OR 95% CI = 0.96, 0.62–1.48, p = 0.86), parents born in non-English speaking countries (OR 95% CI = 0.98, 0.57–1.69, p = 0.95) and families with indigenous children (0.94, 0.53–1.69, p = 0.85). However there was an under-representation of one parent families (OR 95% CI = 0.52, 0.38–0.71, p = 0.00) in the survey compared to LGA samples.

**Table 1:** Characteristics of the parent's survey sample compared to the population

	Parent's Survey		Population	
	n	% yes	n	% yes

People born overseas	3309	22.0	1105001	21.4
People born overseas in countries where the language spoken is not English	3309	15.3	1105001	15.5
Families with indigenous children	3009	1.4	91990	1.5
Families with one-parent	3009	10.6	91990	18.5

The socio-demographic characteristics of wave 1 and wave 2 survey respondents were compared and were very similar.

### Analysis

Logistic regression analysis was used to examine the impact of Best Start on MCH indicator variables (as the dependent variable) for both routine data and parental surveys. The independent variables were time of data collection and the presence of a Best Start project addressing MCH. The interaction between these two variables was tested in order to assess the intervention effect.

#### MCH participation

The independent variables were the presence of Best Start and year of data collection. The presence of Best Start in an LGA was addressed by reference to the presence or absence of a MCH Best Start project. Best Start sites commenced in January or July 2003. The years compared were the 2001/2002 financial year and the 2004/2005 financial year. The dependent variables were level of participation in 3.5 year Ages and Stages visit.

The analyses controlled for socioeconomic and demographic differences at area not individual level. It also took into account clustering by site. The analyses were conducted in Intercooled Stata version 10.

#### Parent's surveys

The independent variables were the rounds of data collection (2004 and 2006) and whether there were MCH projects (yes and no).

The dependent variables were the survey questions – Seen information about the 3.5 year Ages and Stages visit and Confident a good parent. The Seen information about the 3.5 year Ages and Stages visit would include information in child health records provided to parent's, information sheets, posters and direct reminders. The analyses were conducted taking into account socioeconomic and demographic differences between respondents.

## Results

### MCH participation levels

Rates of attendance at the 3.5 year Ages and Stages visit were lower across the 11 disadvantaged Best Start sites than the rest of the state (see table 2) but these differences were non-significant in multivariate analyses when area level demographic differences were taken into account (see Best Start in table 3). There was also a significant increase in attendance at the 3.5 year Ages and Stages visit in 2004/05 compared to 2001/02 across the state (see 'Year-2004/05 vs 2001/02' table 3). However the increase in attendance was significantly greater at Best Start sites than the rest of the state (see table 2 and 'Best Start *Year' in table 3) suggesting that the intervention had an effect.

**Table 2:** Indicator data – changes in attendance at MCH 3.5 year Ages and Stages visit, 2001/02–2004/05

**Predictors**		**3.5 year Ages and Stages visit**	
		2001/02	2004/05

Best Start	Total n	1,739	1,437
	% attended	37.2%	57.5%

Rest of the state	Total n	45,497	45,953
	% attended	49.3%	56.8%

**Table 3:** Indicator data – Effect of Best Start MCH projects on Attendance at MCH 3.5 year Ages and Stages visit, compared to the rest of the state

**Predictors**	**3.5 year Ages and Stages visit**
	AOR(95%CI)

Best Start	0.65 (0.39–1.08)
Year-2004/05 vs 2001/02	1.35 (1.19–1.54)*
Best Start *Year	1.69 (1.12–2.55)*

* p < 0.05, controlling for area, indigenous status, education, country of birth and proficiency reading English; ^# ^Adjusted odds ratio.

### Parent's surveys

#### Seen information about 3.5 year Ages and Stages visit

Levels of seen information were significantly lower at the end compared to the beginning of the Best Start period across Best Start sites overall (see table 4 and 'wave' in table 5). Levels were no different at Best Start sites with and without MCH projects (see 'MCH projects' in table 5). Parents were more likely to have seen information about MCH attendance at Best Start sites with MCH projects at the end compared to the beginning of the Best Start period indicating an effect of the Best Start intervention (see table 4 and 'MCH*Wave' in table 5).

**Table 4:** Survey data-Changes in MCH indicators at wave 1 compared to wave 2

**Maternal and Child Health**			**Wave 1**	**Wave 2**
Seen information about 3.5 year Ages and Stages visit	No MCH projects	n	382	336
		%	42.2%	32.7%
	
	MCH project	n	956	1186
		%	49.2%	51.0%

Confident a good parent	No MCH projects	n	405	337
		%	95.8%	94.4%
	
	MCH project	n	1234	1480
		%	94.7%	97.0%

**Table 5:** Survey data- The effect of Best Start MCH projects and partnership scores on MCH indicators at wave 1 and wave 2, compared to Best Start sites without MCH projects

	Seen information about 3.5 year Ages and Stages visit	Confident in being a good parent
	**AOR (**95%CI)	**AOR (**95%CI)

	n = 2679	n = 3224

Wave	0.65 (0.54–0.78)	0.78 (0.56–1.09)
MCH projects	1.13 (0.8–1.59)	1.0 (0.79–1.29)
MCH*Wave	1.76 (1.2–2.57)*	1.9 (1.16–3.24)*

AOR- Odds ratio for strongly agree/agree compared to reference neither/disagree strongly disagree adjusted for having a health care card, indigenous status, education, country of birth and proficiency reading English.

*p < 0.05

#### Parental confidence

Levels of seen *Parental confidence *were not different across the Best Start period in Best Start sites overall (see 'wave' in table 5) nor were they different in Best Start sites with and without MCH projects. Parents were more likely to be confident as parents at Best Start sites with MCH projects at the end compared to the beginning of the Best Start period indicating an effect of the Best Start intervention (see table 4 and 'MCH*Wave' in table 5).

## Discussion

Best Start aimed to improve child health outcomes in some of the most socially disadvantaged communities in Victoria through local partnerships and improved service co-ordination. In the three years of the program it was effective in improving the uptake of the MCH 3.5 year Ages and Stages visit. This was independently confirmed in a performance audit undertaken by the Victorian state government. [24] The findings suggest that improvements in access to services in disadvantaged areas can be achieved by area-based interventions which focus on optimising the use of existing resources. They may also suggest that the potential health benefits of area-based interventions might be better assessed by examining steps along the pathway between intervention and outcome.

The 3.5 Ages and Stages visit is particularly crucial in child development because it enables developmental problems to be identified and addressed before children attend school. There was an increase in participation in use of MCH services for 3.5 year Ages and Stages visit over the whole state in the period from 2001/02 to 2004/05. The presence of Best Start significantly improved attendance at the MCH 3.5 year Ages and Stages visit taking into account this normal rate of growth in participation. The results provide evidence for a Best Start effect although it is not clear how generalisable this effect might be. It is possible that Best Start simply amplified an existing groundswell in service use and would not have been as effective in the absence of this overall trend. However the odds of attending at the 3.5 year Ages and Stages visit in Best Start areas in 2004/05 was 70% greater than in comparator areas and time periods, a much greater increase than in the rest of the State.

In assessing the effects of complex intervention, it is important that mechanisms through which the program caused change are identified. [25] The results from the parent's survey suggested that Best Start sites offering a MCH program may have had improved participation in the MCH attendance by improving parent's access to information about MCH and promoting overall parental confidence, more so than in other Best Start sites offering other health or educational programs (but not MCH). [26,27] The first finding supports previous research demonstrating the effectiveness of reminders in increasing childhood immunisation. [16] It further suggests that these results might be expanded to other areas of childhood service use.

The increase in parent's confidence in Best Start areas with MCH interventions could either be the consequence or cause of greater participation in the 3.5 year Ages and Stages visit. There is evidence that low parental confidence is a barrier to health service use so improved confidence through health promotion activities may have resulted in improved participation in the 3.5 year Ages and Stages visit. [17] There is also growing evidence that interventions can improve parenting. [28,29] Improving parenting is one of the aims of the MCH program so it is also possible that improved attendance at the 3.5 year Ages and Stages visit increased parental confidence. We are unable to directly link changes in attitudes with exposure to Best Start or changes in service use at an individual level as this data was not available. Consequently we can not disentangle the causal relationships between different variables. However the very high levels of parental confidence suggest that a targeted rather than a population based interventions might be most effective in improving parental confidence.

The study attributes exposure to the intervention at an area level. This is consistent with previous research [1] and appropriate given that almost all parents are exposed to MCH services when their children are first born. While this approach may be criticised for perpetuating the ecological fallacy, alternative approaches to analysing the results of area-based interventions have been criticised for being overly atomistic. [30] Other studies have identified a socioeconomic gradient in the impact of area-based interventions on child and parental outcomes. We could not assess the presence or absence of such a gradient because demographic data were only available at an area level for the analysis of health service use. Cluster randomisation was precluded in this study because sites were preselected by DHS. The Best Start survey sample had an under representation of one parent families compared to the rest of the LGA it is not clear how this might have affected the results.

## Conclusion

Best Start was associated with improved access to MCH 3.5 year Ages and Stages visit; it was also associated with improved access to information about visits and parental confidence. The results suggest that area-based initiatives may be effective in improving access to services with minimal additional resources. The results also suggest that changes in health service may be a positive and overlooked benefit of area-based interventions.

## Abbreviations

MCH: Maternal and Child Health; DHS: Department of Human Services.

## Competing interests

The authors declare that they have no competing interests.

## Authors' contributions

MK, DD, AN and BR contributed to the design of the study. MK drafted the paper and completed the statistical analysis. PF co-ordinated data collection and contributed to the statistical analysis. All authors read and approved the paper.

## Pre-publication history

The pre-publication history for this paper can be accessed here:


